# Adjusting the analytical aperture: propositions for an integrated approach to the social study of reproductive technologies

**DOI:** 10.1057/s41292-021-00240-w

**Published:** 2021-08-19

**Authors:** Victoria Boydell, Katharine Dow

**Affiliations:** 1 The Graduate Institute, Geneva (IHEID), Chemin Eugène-Rigot 2A, 1202 Geneva, Switzerland; 2 University of Cambridge, 16 Mill Lane, Cambridge, CB2 1SB UK

**Keywords:** Reproductive technologies, Gender, Life course, Interdisciplinarity, Reproductive justice

## Abstract

The ever-expanding availability of reproductive technologies, the continued roll-out of ‘family planning’ and maternity services across low- and middle-income settings and the rapid development of the fertility industry mean that it is more likely than ever that individuals, especially women and gender non-conforming people, will engage with more than one RT at some point in their life. These multiple engagements with RTs will affect users’ expectations and uptake, as well as the technologies’ availability, commercial success, ethical status and social meanings. We argue that an integrated approach to the study of RTs and their users not only makes for better research, but also more politically conscious research, which questions some of the ideological precepts that have led to reproduction being parcelled out into biomedical specialisations and a disproportionate focus on particular forms of reproduction in particular disciplines within public health and social science research. We offer this article as part of a wider movement in the study of reproduction and reproductive technologies, which takes inspiration from the reproductive justice framework to address forms of exclusion, discrimination and stratification that are perpetuated in the development and application of reproductive technologies *and* the ways in which they are studied and theorised.

## Introduction

It is high time that studies of reproductive technologies stop assuming that their central artefacts of interest are to be found only in the biomedical clinic.

- Donna Haraway, 1997

In *Disciplining Reproduction*, Adele Clarke (1998) sensitised us to the ‘disciplining effects’ as new fields are formed and how, as these fields consolidate their intellectual and methodological traditions, inadvertent blind spots emerge. Studies of reproductive technologies started to proliferate in the social sciences soon after IVF, the keystone *assisted* reproductive technology (ART), became more widely available from the 1990 s onwards. These studies built on a long tradition of social scientific studies of reproduction and were closely tied to important feminist critiques of the binaries of nature/culture, female/male, private/public (see Ginsburg 1998; Martin 2001, Floyd-Davis and Sargent 1997).

In one of the most important and influential texts of this time, *Conceiving the New World Order*, Faye Ginsburg and Rayna Rapp (1995) argued that reproduction ought to be at the centre of social theory, rather than a marginalised, feminised add-on to more established fields. Since then, reproduction and fertility have in many ways moved further up the billing in public and academic discourse, whether in relation to the proliferation of assisted reproductive technologies, concerns about ultra-low fertility in East Asia, the intensified medicalisation of pregnancy, birth and parenting, or reheated debates about ‘overpopulation’ in relation to climate change. This is therefore an apposite time to train our focus onto the ‘disciplining effects’ of the studies of RTs themselves.

At present, sophisticated reproductive technologies, specialist clinical knowledge and commissioning orthodoxies are driving single-issue approaches that typically focus on one aspect of reproduction at a time. Yet, with increasing technological developments, reproductive healthcare has been gradually divided into ever more specialised biomedical and clinical fields that deal exclusively with one aspect of reproduction, such as fertilisation, or its prevention. Alongside this, some important issues which do not fit these assumptions have been neglected. The fragmentation of service provision and clinical research has parallels across research in public health and the social sciences, as well as in the fields of law and social policy. This means that researchers and health professionals concerned with reproductive health are at risk of missing the intersections between different technologies and services across the life course. Here we propose ways to look at reproductive technologies across time and space.

This article comes out of a workshop sponsored by the Brocher Foundation in Switzerland. At that workshop, Lisa Harris, a professor of obstetrics and gynecology at the University of Michigan and practicing clinician, who also wrote her PhD thesis on the history of IVF in the USA, told us a story from early in her clinical practice. While looking at her notes before inviting a patient into her office, she saw that the patient was concerned that she might be infertile. Harris told us how, in the moments between reading the notes and ushering the patient in, she had formed a mental picture of her, as a smartly-dressed, middle-or upper-class white woman with a busy and important job and was surprised when instead a casually-dressed black woman in her twenties entered the room. As Harris noted, her own mental picture reflects a common association between infertility, ‘career women’ and delayed motherhood in American culture. As she went on to explain, this association is not only inaccurate, since in fact higher levels of infertility are found amongst low-income women in the USA (Bell 2014), but also reflects reproductive stratification (Colen 1995) in the US, fuelled by insidious ideas about whose fertility should be valued and valorised, which both reflect and reproduce intersecting inequalities of race, class, gender, sexuality and disability. Inspired by this telling example from clinical practice, in this article we ask, what precepts, assumptions and ways of working have built up around the study of reproductive technologies? What are the norms of this lively and dynamic field?

We offer this article as a complement to a wider set of reflections in the burgeoning field of studies of reproductive technologies which are aimed at addressing forms of exclusion, discrimination and stratification that are perpetuated in both the application of RTs *and* the ways in which they are studied (Bell 2014; Rudrappa 2015; Valdez and Deomampo 2019, Bhatia et al 2019, Davis 2019; Benjamin 2019; Bridges 2011; Roberts 2017). We agree with Ginsburg and Rapp’s (1995) foundational argument about moving reproduction to the centre of social theory. As they also recognised, we should ensure this happens in a way that is constantly alert to the hegemonic interests and mainstream approaches of our broader disciplines and the power structures of the societies in which we work. We must also remain attentive to who is able and supported to do this research.

We take inspiration from the work of scholars and activists in the reproductive justice movement, which draws on intersectional theory, black feminist thought, decolonial scholarship and queer studies, who argue for a broader view of reproduction and reproductive rights that is less focussed on single events or narrow approaches such as ‘access’ to particular technologies like abortion (see Ross and Solinger 2017; Crenshaw 2017). Reproductive justice exhorts us to, instead, look at how reproductive experiences and processes are shaped by the power structures in which they take place, to be attentive to the injustices which determine whose reproduction is valued or discounted and to account for the many different forms of labour that go into conceiving, birthing and raising a child and the myriad people who play a part in this.

## An integrated approach

In this article, we argue for, and invite, a more integrated approach to the study of reproductive technologies. By an integrated approach, we mean that RTs should be studied more holistically and contextually. This is an invitation to turn the analytical gaze back on the methodologies and precepts that have built up around the study of reproductive technologies.

Historians of science and medicine have discussed the prioritisation of visualisation for ways of knowing within science and medicine (see, for example, Landecker 2013; Keller 2003; Daston 2008) and feminist scholars have noted the implications of visualising technologies for reproductive politics (Petchesky and Judd 1998). As these scholars point out, the visual is the preeminent sense in Western epistemology, associated with evidence, knowledge and veracity. Reproductive technologies have often been described by scholars as a ‘lens’, expressing a common assumption that casting light on a particular technology or patient experience can, in Marilyn Strathern’s (1992) terms, ‘literalise’ the deep social and cultural meanings of reproduction, kinship, gender, nature and much more besides in that particular context. Visual metaphors abound in the English language and so, in this article, we use this idiom knowingly to suggest that we might adjust the aperture (see also Almeling 2020) in order to scrutinise our *analytic lenses*.

We recognise that we have our own blindspots,^1^ so, although we offer some suggestions for how to study RTs in a more integrated way, there will be many others that we have not anticipated, and we welcome them. In this article, we suggest that an integrated approach could mean explicitly and actively considering different RTs together and/or studying them within a life course, whether of the technologies themselves and/or their users. We should remain attentive to the reasons that users of RTs give for taking up or rejecting particularly technologies, whilst also recognising both the way in which reproduction continues to be stratified (Colen 1995) and individuals’ agency and acts of resistance in ‘real world’ usage of these technologies. Taking more comparative, historicised and contextual approaches to RTs, we argue, allows for a broader overview of the logics that drive the production, promotion and use of these technologies, offering opportunities to identify (dis)continuities between them and to critically assess their efficacy, value and meanings. This is perhaps especially pressing at a time in which the fertility industry is booming, often outpacing the societal, ethical and legal deliberations which might curb its excesses or, even, point its architects and practitioners in the direction of as yet unimagined and unmet needs.

Katharine Dow (2016) has called for a contextualised analysis of reproduction, arguing that since reproduction is not in reality relegated to the private or domestic domain, we need to ensure that we study it as a part of life, tracing the imaginaries, values and principles that structure reproduction, as well as how reproductive experiences shape other parts of social, political and ethical life. Here the focus is on understanding reproduction as part of the larger social context that frames such experiences and decisions (Briggs 2017). There is much to be learnt from studies of RTs that focus on singular reproductive events in people’s lives, which have thrived over the last few decades. An exemplar would be Sarah Franklin’s (1997) *Embodied Progress*, at once a focussed empirical study of British people’s experiences of IVF in the early days of that assisted reproductive technology, an analysis of how reproduction is shaped by political, economic and social forces from gender inequality to government science policies, and a critical intervention into a longstanding debate about reproductive knowledge within anthropology.

We do not suggest that reproductive scholars abandon studying specific technologies or even specific users of particular technologies, but simply remind them that, as Franklin’s body of work shows so well, no technology, or patient, exists singularly. Rather, we argue that it is time to reflect on what we have learnt more broadly, with a view to refining and building theoretical contributions to the subfield and to the broader disciplines in which these studies take place. We are contributing to a time-honoured call to study reproductive technologies from multiple perspectives, in context and with different lenses (Ginsburg and Rapp 1995; Almeling 2015, 2020). We go beyond this by noting that we need to more precisely locate technologies in time and space: technologies have their own interrelated histories and people experience multiple technologies within and across the life course.

In a recent example, Sharmila Rudrappa (2015) has demonstrated the importance of time and space in understanding contemporary uses of reproductive technologies. She has argued for a different understanding of surrogacy in India, which takes account of the ‘techno-pastoral’ imaginary prevalent in the country’s colonial and postcolonial history. In this imaginary, Rudrappa argues, surrogacy is facilitated by a historic sense that working-class women are akin to land, so that they appear available to technocratic interventions which harvest ‘products’ and profits. Rudrappa’s account shows the value of taking a broader view of surrogacy, a practice that has often been treated in public discourse as inherently novel, bizarre and highly technologized. By linking contemporary surrogacy practice with important historical events from the South Indian famine of 1875–1876 to the Green Revolution, and the way they shaped perceptions and management of fertility, Rudrappa in a sense reintegrates surrogacy into the context in which it is practiced.

Social scientists working with white, middle-class heterosexual cis women affected by infertility have often noted that some of their research participants remarked wistfully on how they spent much of their youth relying on contraception to prevent an unplanned pregnancy only to find, when they were ready to have a child, that they were in fact unlikely to achieve a pregnancy without medical intervention. This common story is a reminder that many people—of any gender or sexuality—think about and encounter multiple RTs throughout their lifetimes, whether that be a couple which conceives a child in their early adulthood subsequently opting for a long-acting reversible contraceptive method and then using IVF to conceive a child later in life, someone with HIV+ status who takes anti-retroviral medication and uses sperm washing to conceive a child, or a heterosexual woman who uses condoms and menstrual tracker apps to prevent an unplanned pregnancy in her thirties before using IVF to try and conceive a child with her previously frozen eggs in her forties. These hypothetical scenarios, which all point to the unruly bodies and messy realities of people’s lives, relationships and reproductive trajectories, underline the importance of recognising that RTs are not limited to biomedical clinics, as Haraway contended over 20 years ago, nor are they reducible to the logics that govern those clinics. RTs do not exist in isolation, just as their users do not; they are multiply relational technologies.

Studies of reproductive technologies need to not only document patients’ experiences, but also examine why particular technologies are and are not developed, made available and to whom and under which circumstances. We think it is important for reproductive scholars to consider whether focussing on specific technologies (and therefore not others) obscures the inequalities of access to RTs *of all kinds* both across the world and within particular countries. We are calling for greater oversight over different reproductive technologies by looking not only at the life courses of technologies’ users, but also of the technologies themselves, the historical contexts in which they emerge and who their likely users are imagined to be.

An integrated approach looks at RTs across time and space, tracing the kinds of connections, absences and assumptions that emerge. We need to explore how the aggregation of technologies is changing, or reproducing, values, norms and rationalities around reproduction, and how it is studied. RTs work, and can be studied, across different scales of time and space, and there are different ways to tackle this. For example, within a person’s biography—considering how life courses are structured through reproduction and the use, or refusal, of RTs. We could also trace the biographies of specific reproductive technologies over time. Another scale would take a comparative approach, including methodologies that actively study a combination of technologies together or seek to understand the relationships between them. Taking an integrated approach means considering the complex interactions between different RTs, their users and providers, offering new perspectives on what particular RTs have in common and where they diverge, in terms of their usage, promotion and development and their social, ethical and political meanings.

We think it is important to look across RTs and to help build dialogue between scholars who work on different aspects of reproductive life, not only from an empirical perspective, but also because otherwise studies of reproduction are in danger of missing, and thereby reproducing, the kinds of power structures that can constrain reproductive life. How can we ensure that specific reproductive technologies are not siloed into particular disciplinary traditions or treated in ways that can (inadvertently) perpetuate reproductive stratifications? Drawing on exemplary studies of RTs and the insights of intersectional feminism and reproductive justice, we insist that reproduction and reproductive technologies can only be understood in their broader personal and political contexts, so as to guard against what Adele Clarke (1998) identified as the constraints imposed by traditional disciplinary boundaries. Indeed, this complex relationship between RTs and the contexts in which they operate—which has often been conceived as a ‘lens’—is why they first came to the attention of social scientists, and we should not lose sight of this.

## Reproductive technologies

In her review of studies of reproduction, Rene Almeling suggests this definition of reproduction: “Reproduction is the biological and social process of having or not having children” (2015, p. 430; see also Murphy 2012). She suggests this in contrast to the implicit understanding of reproduction as a series of events inside women’s bodies that, as she identifies, is characteristic of orthodox Euro-American thinking and clinical rationales. Theorising reproduction as a situated process requires that scholars specify what is (and is not) common to such disparate events as conception and contraception, pregnancy and abortion, birth and infertility. Moreover, as Almeling says, conceptualising reproduction as a process broadens the scope beyond women, making it possible to include men’s bodies and experiences (2015, p. 430)—and, we would add, trans and non-binary experiences—and thereby a more holistic understanding of the array of biological and social processes that shape reproduction, from bodies and kinship to laws and markets.

We live in an era of unprecedented ‘reproductive control’ (Franklin 2013) though it remains largely out of reach for most. There are innumerable ways we can intervene in reproductive processes, using a range of what Sallie Han (2013) has called ordinary and extraordinary technologies. Given the diversity of RTs available, it is useful to define what we mean by technology. We use the term *technology* broadly to include pharmaceuticals, devices and digital applications, clinical and diagnostic procedures. Lock and Nguyen (2018, p. 18) remind us that “Biomedical technologies are, of course, designed expressly to facilitate human intervention into the workings of the body in health and illness; in implementation they change us, and, even as they themselves are constantly modified, they change the world in which we live”. Technologies are inextricably linked, tied together through Enlightenment thinking about the mastery of nature and continual progress (Haraway 1997; Strathern 1992, 1994). The new biomedical technologies are bound up with a political economy of aspiration and hope (Novas 2001; Franklin 1997). Nayantara Sheoran (2015, p. 253) has aptly characterised this as “the promissory emancipatory potential of these new medical technologies*”;* as her work shows, in local realities, though, this is often limited to all but a privileged few.

A reproductive justice framing also reminds us that reproductive technologies have both emancipatory and repressive genealogies. It is by now well established that reproductive technologies have historically been fuelled by eugenic agendas and that in many ways it is the financial and political capital from populationist campaigns, wedded with first- and second-wave feminist activism, that made a range of RTs (and not only contraceptives and abortion) available in the first place (Roberts 2017). Dorothy Roberts’ point that “America’s recent eugenic past should serve as a warning of the dangerous potential inherent in the notion that social problems are caused by reproduction and can be cured by population control” (2017, p. 71) sadly holds true over 20 years after the first edition of *Killing the Black Body* (see also Bridges 2011; Murphy 2017). Reproductive justice scholars and activists have shown how eugenic ideologies persist, shaping how many think about reproduction, whether and how it should be enabled by particular technologies and who their proper users are. In other words, reproductive technologies do not only facilitate the reproductive ‘choices’, needs or desires of their users to effect or prevent reproduction, but are also technologies that reproduce intersecting inequalities; they are reproductive technologies in more than one sense of the term.

Here we draw on Lock and Nguyen’s (2018, p. 20) formulation that “technologies should be understood as both produced through culture and as productive of culture”. Technologies and their routines are defined and embedded within the epistemic values and normative frameworks of the biomedical practitioners that develop and provide them and the national governmental, international bodies and commercial entities that regulate access to them. The cultural scripts of developers shape what the technologies can be and do, yet these in-built precepts about technologies are not necessarily or neatly replicated in local practices and experiences (Maffi 2016; Franklin and Inhorn 2016). Rather, the provision and use of technologies, as many have argued, are heterogeneous and fluctuating (Hardon and Moyer 2014; Beaudevin and Pordie 2016; Maffi 2016; Sanabria 2016).

We consider any reproductive technology one that works on “the biological and social process of having or not having children” (Almeling 2015, p. 430) and this encompasses conception and contraception, pregnancy and abortion, birth and infertility. This is the intended meaning when we use the term in the article. We recognise the technological bias in this definition but argue that it is a realistic reflection of the role of medicine and technology in an ever-increasing number of people’s lives and note that not all reproductive technologies are ‘high tech’. Nowadays RTs are widely known and/or available and have become normalised and routinised in high-, middle- and low-income countries (Franklin 2013; Wahlberg 2016). In many places, the use of RTs has become expected to such an extent that reproductive ‘choice’, facilitated by technology, is experienced as an obligation and cultural imperative (Solinger 2001; Russel et al. 2000; Murphy 2012; Gammeltoft and Wahlberg 2014; Franklin and Roberts 2006; Sandelowski 1993; Strathern 1994; Wahlberg 2008; see also Emre 2018).

## Studies of reproductive technologies in the social sciences

There is nothing wildly new about people trying to determine when, where and how they reproduce. As Rosalind Petchesky and Judd (1998) reminds us, women (in particular) attempt to control their reproductive lives, often at great risk to themselves: they “not only cope patiently with meagre resources and intransigent cultural and social barriers…but defy the tradition of female passivity: manoeuvring around, subverting, bending, or sometimes directly challenging those barriers.” Yet what is different in ‘the age of reproductive control’ is how routine the (bio)medicalisation of reproduction has become. Reproduction is increasingly subject to a range of professional and techno-scientific interventions which have not only detached reproduction from sex and intimacy, but separated reproduction into discrete events that are ‘parcelled out’ into distinct clinical specialisations with their own expertise, diagnostics, treatments and procedures. Reproduction, like parenting, has become an “expertise-saturated, policy-focussed and commercially fuelled area of social life” (Faircloth and Gurtin 2017, p. 986).

Alongside the rapid expansion of RTs and their application in clinical practice, social research and scholarship around their development, use and meanings has grown. A thriving interdisciplinary field has emerged that has both tracked and attempted to unpick the (bio)medicalisation of reproductive experience. This field lies at the intersection of feminism, science and technology studies, medical sociology and medical anthropology (Martin 2012). Hampshire and Simpson (2015) provide us with a useful characterisation of how the field has evolved, particularly in relation to assisted reproductive technologies. The first phase, between the 1980s and 1990s, provided ground-breaking accounts of assisted reproduction in the realms of kinship, family and reproduction that focussed primarily on white, middle-class women in the Global North (Strathern 1992; Franklin 1997; Thompson 2005). The second phase, from the late 1990s onwards, captured accounts of the diverse forms assisted reproductive technologies take in a range of settings, such as India (Bharadwaj 2006) and Egypt, though often among elites. We suggest that more recently there has been a focus on reflecting the experiences of more marginalised populations, captured in the work of Valdez and Deomampo (2019) (see also, for example, Bell 2014; Murphy 2017; Rudrappa 2015; Bhatia et al 2019; Davis 2019; Blell 2017).

There are important insights to be gained from these studies of reproductive technologies and we highlight some key precepts from this industrious field below. However, as noted at the outset, as any field or discipline grows, and focuses on particularly areas, it also inevitably and inadvertently develops certain empirical and analytical blindspots, as the most recent wave of studies also points out. We propose, therefore, that it is time to reflect on what studies of reproductive technologies have achieved while also critically examining their precepts, legacy and future potential. To identify the central themes, we read several recent papers that review the field of reproductive technology studies, or particular elements of it, to draw out the common threads (Birenbaum-Carmeli and Inhorn 2008; Smietana et al. 2018; Gammeltoft and Wahlberg 2014; Franklin and Ragone 2006; Franklin and Inhorn 2016; Faircloth and Gurtin 2017; Almeling 2014; Valdez and Deomampo 2019; Bhatia et al 2019). We were looking for existing thinking on integrated approaches, particularly how scholars have documented people’s experiences of the aggregation of reproductive technologies and/or how they have examined reproductive technologies across time.

### (Bio)medicalisation

This relates to the creation of medical specialisms that work on a universalised body with professional experts controlling specialised medical knowledge and interventions (Cooper and Waldby 2014; Clarke et al. 2003; Rose 2007). People are increasingly interacting and engaging with biomedical knowledge and practice (as well as practising it) in relation to their reproduction. Studies that focus on biomedicalisation—for example, Rapp (1999), Thompson (2005), and Davis (2019)—provide vivid examples of how women (in particular) actively engage with medical language and practice to achieve their own ends, however ambivalent those might be. Examining overarching trends like biomedicalisation is helpful in that it allows us to get a broader picture of RTs and how they are linked up with particular institutions and epistemologies, though we would urge that it should be approached through an intersectional lens that takes into account the full range of inequalities that neoliberalism perpetuates.

### Normalisation and naturalisation

Encounters with reproductive technologies reflect and shape ideas about what is normal and what is natural. Drawing on presumed universal laws and statistical constructs, ideas of what is normal in biomedicine are tied up with ideas about how things ought to be and often rooted in ideologies of nature and biology (Fausto-Sterling 2000). Research by social scientists has allowed us to better understand processes of normalisation and naturalisation and how these ideas remain powerful (Dow and Boydell 2018; Franklin 1997; Strathern 1994; Pralat 2018). An integrated lens retains this critical focus on relationships between norms and ideas of the natural as well as begging questions about whether our ideas about what is ‘normal’ and ‘natural’ transcend our engagement with different reproductive technologies or whether they change over time depending on experience and context.

### Individualisation

In the neoliberal worldview that has influenced the development of biomedicalisation, people are primarily conceptualised as individuals who have a moral responsibility to optimise themselves (Rose and Novas 2003; Rose 2007; Baldwin 2018, 2019). Reproductive healthcare becomes a personal moral responsibility to be fulfilled with improved self-surveillance and norms and standards made possible by available technoscientific interventions. This is particularly notable in ethnographic studies of contraception where the technology itself is expected to instigate an ideational change from traditional fatalism to modern rational and responsible self-regulated individuals, that is, ‘modern subjects’ (Collier 1997; Paxson Paxson 2002, 2004, Greenhalgh 1995). An integrated approach would further this point by asking how individuals and their partners use different technologies at once or at different times, what transformative effects this has and how the goals of optimisation change over time.

### Resistance and agency

There are ongoing debates about how (bio)medicalisation relates to subjectivities—whether extreme over-deterministic self-governance (Rose 2007) or more of a co-constituted interaction between people and biomedical technologies (Latour and Woolgar 1979; Lock 2017). Scholars illustrate how scientific knowledge is filtered, interpreted and evaluated through people’s own lenses (Rapp 1999; Greenhalgh et al. 2014; Timmermans and Berg 2010; Martin 2001). More than that, people find different ways to resist or subvert biomedical knowledge, opting and/or refusing to reproduce can be a site of resistance (Thompson 2005; Martin 2001; Davis 2019; Sheoran 2015). Considering the temporal dimensions of RTs as part of an integrated approach could allow scholars to examine how resistance and agency change over time and whether certain technologies are more or less likely to be resisted.

### Political economy of reproduction

The fertility industry is booming, with huge profit margins. For scholars of reproduction in the Global North, this is particularly notable since many contraceptive and assisted reproductive technologies were developed in what might now be called a ‘public-private partnership’ and this has led to the establishment of a thriving private healthcare sector even in countries with public healthcare systems, including for contraception, antenatal and maternity care. With the ever-expanding horizon of RTs, reproduction has been opened up to political and commercial forces in new ways, bearing down on sexual and reproductive choices, enlarging some people’s options whilst (further) stratifying access to and use of (particular) RTs for others. This is perhaps most evident in the recent work around commercial surrogacy and the power asymmetries surrounding transnational reproductive care (Deomampo 2016; Schurr 2018, 2019; Sarojini et al. 2011; Twine 2015), reproductive substances (Nahman 2011; Kroløkke 2018) and reproductive data (van de Weil 2020a, b). Kroløkke (2018), for example, traces different reproductive matter and fluids to reflect upon the global flows of cultural values, laws, exchange systems, and ethics in a globalised tissue economy. Comparing and contrasting access to different technologies in this way can bring intersecting inequalities and political and economic agendas into stark relief.

### Stratified reproduction

Shellee Colen’s (1995) concept has proved to be a perennially helpful framework for understanding the ways in which people are sorted into groups whose reproduction is differently valued and supported both by broader society and medical infrastructures, in line with intersecting inequalities of race, class, gender identity, religion, (dis)ability and sexuality. This different valuation of reproduction is readily apparent in the clinical trials of hormonal oral contraceptives in Puerto Rico and the mandatory sterilisation campaigns in India (Watkins 1998). Similarly, we find that the approach of reproductive justice, which criticises the reproductive rights movement for its focus on ‘choice’ and access to abortion while many people are simultaneously being denied a right to have children or to care for the ones they already have, demonstrates the value of our aim here of encouraging dialogue across RTs (Ross and Solinger 2017; Smietana et al. 2018). The juxtapostions created through a more integrated approach, particularly comparing technology across social divides (see Bell 2014; Davis 2019), can generate important insights into the generation and perpetuation of stratified reproduction.

Alongside the recurrent themes identified in this brief review of studies of reproductive technologies, we have also traced some enduring conversations in the literature, including on the topics of change, gender and reproductive labour. The introduction of new technologies changes the individual and collective lives of those who use or imagine them, yet there is much debate about how this change comes about. More technologically deterministic arguments suggest that such change is an effect of the technology itself, yet many have argued that this approach does not adequately consider how people and their local moral economies shape and influence how technologies are used, adopted, understood and shared (MacKenzie and Wajcman 1999; Hardon and Moyer 2014; Beaudevin and Pordie 2016). Reproductive scholars have shown that expanded access to RTs can represent significant culture change (Birenbaum-Carmeli and Inhorn 2008; Franklin 2013; Ginsburg 1998; Paxson 2004; Collier 1997), not least in opening up conversations about topics that are in many places taboo (Inhorn et al 2017; Gürtin 2016; Wainwright et al 2016). RTs can lead to a fragmentation and denaturalisation of reproduction, in which, despite apparently ‘chasing the blood tie’ (Ragoné 1994), the meanings of kinship and the relationship between nature and culture or science and society are profoundly altered (Deomampo 2016; Strathern 1992; Franklin 1997; Franklin and Roberts 2006). Yet empirical studies have repeatedly shown that technologies are not necessarily understood, used and imagined as revolutionary, but rather (re-)embedded within existing cultural logics, incorporated into existing idioms and even reinforcing acceptable social arrangements and norms, though often in previously unimaginable forms (Bhatia 2018; Gammeltoft and Wahlberg 2014; Franklin and Ragone 2006; Ginsburg 1998; Paxson 2004; Collier 1997; Franklin 2006, 2013; Franklin and Inhorn 2016; Thompson 2005; Murphy 2017).

These norms, particularly around motherhood and fatherhood and femininity and masculinity, retain a powerful hold, including in avowedly modern and secular societies and even when these technologies are being used by single and/or LGBTQ+ parents. These tensions between novelty and normativity are well illustrated in the burgeoning of studies of LGBTQ+ reproduction, which provide not only important empirical data about ‘alternative’ forms of parenting and how LGBTQ+ people are able (if indeed they are) to access RTs, but another way into recognising and understanding the social, cultural and political forces that shape reproduction more generally (Nordqvist 2014; Walks 2008; Mackenzie 2013). Robert Pralat (2018) has shown that for some gay, lesbian and bisexual people who have come of age at a time in which gay marriage, adoption and fertility treatments have become available in some contexts, this does not necessarily translate straightforwardly into ready take-up of these options. In fact, the costs and realities of assisted reproductive technologies, in particular, may cause significant ethical dilemmas for LGBTQ+ (intended) parents, perhaps especially when surrogacy is involved (Smietana et al. 2018). Taking account of LGBTQ+ reproduction is imperative in itself in the interests of inclusivity, but an integrated approach reminds us also that individuals’ sexual identities and relationships can shift over time and so studies of RT should also account for this.

Ginsburg’s (1998, p. 213) pioneering work around abortion suggested that the womb is “the last unambiguous symbol of [an] exclusive female arena”. People who identify as women are, still, generally considered responsible for all aspects of reproduction. From self-administering hormone injections before egg harvesting, to remembering to take contraceptive pills, to logging pregnancy experiences or charting menstrual cycles through apps, to taking time out of work to attend clinical appointments, to using breastpumps to maximise their child’s nutrition and doing the majority of childcare once a child is born, women, trans men and non-binary people bear the lion’s share of embodied reproductive labour. This labour might appear similar from the medical point of view, but gender non-conforming people, like other queer parents, are of course far more likely to be subject to greater scrutiny and criticism than cis and/or heterosexual women and this will have effects on their embodied experience and (access to) medical care.

Many studies document the efforts required to access and use RTs as a new form of reproductive labour that intersects with evolving feminist scholarship (Thompson 2005; Martin 2001; Baldwin 2019; Davis 2019; Cooper and Waldby 2014). This gendered embodied labour is rationalised through a ‘naturalising’ logic (Faircloth and Gurtin 2017). This speaks to the persistent power of normative assumptions about gender around reproduction in the life course (Becker 1999, 2000; Faircloth and Gurtin 2017; Hovav 2020). As Almeling identifies, there is a tendency to look at RTs as discrete events occurring in women’s bodies and/or clinical settings and as largely affecting women. As she notes, while it may be empirically true that those who identify as women are the main consumers of these technologies, this does little to complicate the assumption that reproduction is women’s domain—and accordingly undervalued—nor does it reflect reproduction’s relational nature (see, for example, Inhorn 2003). Kroløkke (2018) and Lock and Franklin (2003) illustrate how reproductive processes are at the centre of commercial biosciences and a source of value and biocapital. Yet despite this commercial potential, new forms of reproductive labour are feminised and marginalised in different ways (Waldby and Cooper 2010; Briggs 2010; Dickenson 2006; Hovav 2019). Some radical voices have used this point to call for a dismantling not only of patriarchy, but also the family and for new recognition given to ‘gestational labour’ (Lewis 2019; Cuboniks 2018; see also Firestone 1970).

Recent feminist scholarship, such as Nancy Fraser’s work (2013), has detailed how socially advantaged women’s ability to take advantage of new opportunities to study, gain educational qualifications, work, gain control over their fertility and explore their sexuality are dependent on disadvantaged women taking on their reproductive and care roles, which is reflected in the recent ethnography of Daisy Deomampo (2016; see also Hooks 2000). In Laura Briggs’ recent book, *How All Politics Became Reproductive Politics*, she helpfully connects the dots of stratified reproduction, ‘structural infertility’, neoliberal economics and assisted reproductive technologies in the contemporary USA. As she points out, as well as worrying about how the contours of race and class are implicated in who provides cells, bodies and labour to support fertility treatments, we must also pay attention to how expectations of work, professionalism and career development militate against younger child-bearing for those who seek to enter or stay in the middle-class and, in turn, feed into the expansion and intensification of the fertility industry. As she writes, “This [generation’s] infertility is induced by the labour market—and the toxic health by-products of racial and other inequalities—and it is driving a highly profitable but not very effective ART industry” (2017, p. 112).

As Faircloth and Gurtin (2017) note, the possibilities that are made imaginable with RTs bring with them a host of new decisions, embedded within existing sensibilities, moral norms and social aspirations over their life course, as well as ongoing scrutiny about the ‘choices’ they make. These apparent possibilities are often presented and experienced as individual choices in such a way that obscures the structural inequalities and the background manoeuvrings of industry and government that delimit what are permissible choices for people who identify as women (Davis 2019; Orgad 2018). These constrained freedoms have become all the more apparent in the Covid-19 pandemic (Crook 2020).

It is no wonder that this is a time of reproductive anxiety. Personal anxieties are no doubt fuelled by the ongoing heated public debates around areas of reproduction—whether abortion, adolescent pregnancy, infertility or egg-freezing, to name but a few examples (Ginsburg 1998; Cannell 1990; Bharadwaj 2006; van de Wiel 2014). RTs seem to be surrounded by ambivalence and uncertainty, or at least to heighten these emotions, which might already occur in a less technologised scenario. Ambivalence and uncertainty have been acutely articulated in work around selective reproductive technologies and responses to disability and genetic disease including Rayna Rapp’s formative description of women undergoing amniocentesis as ‘moral pioneers’ (Rapp 1999; Gammeltoft 2014), in the experience of infertility and assisted conception (Bharadwaj 2006; 2016) and in attempts to model pregnancy intention (Higgins et al 2012; Jones 2017; Aiken et al 2016). Uncertainty accompanies even the humblest of technologies, for example Ross (2018) describes how home pregnancy test results are filtered through emotional and sociocultural contexts and rather than addressing the unknowns of a possible early pregnancy, positive results exacerbate them. Reading across technologies, we see that there is an optimistic assumption that biomedical technologies are positioned to ‘emancipate’ people from uncertainty in the reproductive process among practitioners and patients. Yet, with pregnancies continuing to elude rational management, diagnostic results open to interpretation, side-effects of contraceptives leading to discontinuation, IVF cycles regularly failing and extended HRT use associated with a range of reproductive cancers, RTs often do not deliver on their promise of control or choice (Ruhl 2002; Granzow 2010).

## Adjusting the analytical aperture: propositions for reading across reproductive technologies

Recent approaches in the study of reproductive technologies illustrate the empirical, and analytical, benefits of contextualised, historicised and comparative methodologies. Franklin and Inhorn (2016) have published an ambitious comparative project that charts the distinctive characteristics of IVF histories in different global contexts, in order to identify changing cultural values, norms and rationalities around (assisted) reproduction. Similarly, Gammeltoft and Wahlberg (2014) and Wahlberg and Gammelotf (2017) have synthesised work examining how different selective reproductive technologies are made available in different contexts and how people perceive and make use of these new possibilities in their lives. Another recent effort to read across sub-fields that is highly relevant to the more integrated approach we advocate here is Faircloth and Gurtin’s (2017) work bringing research on parenting and assisted reproduction into conversation, which highlights connections between reflexivity, gender, expertise and stratification before and after conception and birth.

In their recent double special issue, Valdez and Deomampo examine why the analytic of race is still rarely sufficiently integrated in studies of reproductive technologies. They apply “ethnographic methods in diverse settings to reveal the racism inherent to reproductive health care and policies” (2019, p. 556), looking across different RTs to identify reproductive injustice. Bhatia et al. (2019) have also set out to examine contemporary forms of population control, a logic that categorises people and turns them into numbers to rationalise the use of interventions which reinforce power asymmetries (see also Murphy 2017; Sasser 2018). This includes interventions to have ‘better quality children’ which move through fertility limitation to assisted reproduction. These two collections are inspired by black feminist analysis and the reproductive justice movement, and serve as a corrective to the reproductive rights movement’s singular focus on access to abortion. The reproductive justice framework demonstrates how this singular focus of one form of reproduction obscures the forms of exclusion, discrimination and stratification that continue to be perpetuated in reproduction, science and technology and its study (see also Bell 2014; Davis 2019; Benjamin 2019; Bridges 2011; Roberts 2017). The fact that the editors and authors of these two collections discuss a broad range of reproductive technologies strengthens their analysis, showing how reproduction is stratified in both overt and insidious ways and how this is perpetuated through a lack of attention to pervasive racism and reproductive injustice.

We aim to build upon these recent observations and argue for a far more explicitly integrated approach in the study of reproductive technologies. This requires accounting for the temporal dimensions of reproductive technologies, which in turn reveals the fact that people often experience multiple technologies within and across the life course. As well as identifying the importance of reading across RTs, we wish to make some constructive suggestions for how reproductive scholars might approach reproductive technologies in a more integrated way, by discussing how RTs, like any other technology, can be studied across both temporal and spatial axes.

### Juxtaposition

One way to consider RTs is through comparative studies that juxtapose technologies or approaches. Firstly we may consider how RTs relate to individuals’ life courses and the ways in which different RTs do, and do not, come into play at certain points and in relation to particular people’s experiences and relationships. We began with the simple observation that people tend to engage with multiple technologies at once or within a lifetime. Similarly, in practice, many RTs are associated together, both explicitly and implicitly—for example, foetal screening is often tied up with an assumption of abortion following a positive result (Rapp 1999; Gammeltoft and Wahlberg 2014) and this in itself points to important conversations about disability rights and the legacies of eugenics and population control. We are interested, given the many technologies that have become normalised and more readily available, in how different RTs relate to each other over a person’s life, for example how egg-freezing is tied into contraceptive use and ARTs and seems to both extend fertility as well as assume (future) infertility (Baldwin 2018; Baldwin et al 2019; van de Wiel 2020b). Such an approach reminds us that individuals’ ideas, aspirations, identities and relationships accrue and shift over time and a more longitudinal approach to RTs may better account for this.

We recognise, and applaud, the fact that studies of reproductive technologies within the social sciences tend to be interdisciplinary in their methodologies and theoretical frameworks. Nonetheless, there has historically been a tendency for particular reproductive technologies to be associated with particular disciplines, so contraceptives still tend to get more attention in disciplines such as public health, while ARTs have held a particular fascination for anthropologists, sociologists and cultural studies scholars. This leads us to ask how these disciplinary clusters shape the ways in which these technologies are understood within academic research—and what effects this might have on users’ experiences. Taking an integrated approach could mean researchers studying unexpected topics, such as a scholar in public health looking at IVF provision in a low-resource setting. It could also mean fruitful new collaborations that transgress these silos: what if, for example, a public health researcher studying teen pregnancy teamed up with a cultural studies scholar analysing egg freezing to build new understandings of reproductive ageing?

It is also critical to look at the same technology across social groups, to bring inequities to the surface. In her ethnography of experiences of premature birth amongst black women in America, Dána-Ain Davis (2019) notes the uniqueness of her topic, in its focus on black women of higher socio-economic status. As Davis explains, many public commentators assume that the high levels of maternal morbidity and mortality amongst black American women is due to lower incomes and/or poor personal choices. In fact, black women with higher incomes, high levels of education and professional status are still more likely to have premature births than poorer white women (2019, p. 9). Given this, Davis makes a compelling case that the missing explanatory factor is medical racism, which she relates to longstanding racist stereotypes about black women being hardy and stoical, the kinds of diseases and conditions black women are likely to have (or not have) and whether they are reliable narrators of their own experience.

Although there is increasing interdisciplinarity in reproductive studies, it is still quite rare to see studies of two or more RTs together, or of people who use one RT alongside those who reject it. To take one example, many RTs are linked through the biochemical manipulation of synthetic ‘hormones’—preventing or promoting ovulation for example—and hormones are attributed with the ability to change emotional and physical states (Sanabria 2016; Oudshoorn 1994; Fausto-Sterling 2000). Ideas about hormones may cut across experiences of reproductive processes and commute from one technology to another. Recognising the similarities and differences between ingesting synthetic hormones, which might well be made by the same company, to achieve a gender transition or to prevent conception of an embryo, suggests a means to achieve a more complex engagement with the realities of people’s reproductive and relational lives.

### Life course(s)

In understanding people’s histories and experiences, we must address anticipated futures but also how early experiences set the stage for future ones (Elder 2003). These experiences happen within specific historical moments in which people’s lives unfold. This suggests that the life course is cumulative and there are benefits to looking at the whole rather than singular reproductive episodes or technological engagements. We must remember that, just as there is no universal life course, reproducing and preventing reproduction each make sense at different times in people’s lives—and, importantly, there are many who never wish to reproduce at all.

Thinking about the different RTs that a person might encounter in a lifetime suggests the value of more longitudinal research on RTs themselves. RTs have linked histories, genealogies and biographies. Both RTs and their users, or refusers, exist in specific historical contexts and the technologies which are available, or not, at that time say much about that particular moment. In building global historical narratives of IVF, Thompson (2016) and Wahlberg (2016) have reflected on how IVF was closely intertwined with policies and ideologies around other RTs. Developmental biology, which provided the scientific basis for ARTs in humans, was originally aimed at improving medical contraceptives as part of a broader concern about managing population size in low-resource settings (Hartmann 2016). Further, as Adele Clarke (1998) has discussed, RTs in humans developed alongside agricultural research and breeding technologies, yet those studies that address this, while often fascinating, are rare (see Haraway 1997; Cassidy 2002; Friese 2013; Franklin 2007).

Reproduction and time are perhaps most obviously linked through ideas about timing, planning and decision-making. Early behavioural theories of contraceptive practice suggest linear change from individual intention formation to enacting a behaviour, yet research increasingly shows that contraceptive decisions are regularly revised with new experiences and knowledge. Over time the experience of using contraception accrues and decisions are not based on a distant future, but rather in more immediate concerns (Downey et al 2017). Yet the meanings and values of contraceptives are influenced by knowledge and experiences of, not only other contraceptive methods, but also other RTs, whether it be abortions, egg freezing or medicalised childbirth. There has been much emphasis on the anticipatory dimension of RTs, with decisions in the present projecting forward onto future reproductive decisions far in advance, suggestive of reflexive and forward thinking (Adams et al. 2009). One example is the emergence of pre-conception/pre-pregnancy care, ranging from selective reproductive technologies and screening to immunisations and contraceptives (Waggoner 2017). Reproduction and time are also bound up with ideas about ageing and the effects of time on the body. We see this perhaps most clearly through the example of egg-freezing, which is typically associated with a range of RTs, from contraceptives to prevent pregnancy at the ‘wrong’ time, to the use of IVF, perhaps using frozen sperm, to achieve conception and, if that is achieved, most likely, a medicalised birth (van de Wiel 2020b).

When we look at RTs across the life course, the intersections and interactions between gender and age become clear. Susan Pickard (2018, p. 7) argues that there is a “gendered structure of temporality” that generates time anxiety and age-related scrutiny of women. For women the biological clock is the centre piece of this awkward conjoining of two rather different agendas, controlling women’s ability to challenge and detach themselves from the age and gender system. The RTs associated with particular age groups, such as contraceptives and human papillomavirus vaccine for adolescents, reflect assumptions about how age, gender and reproduction (should) intersect. Driven by some implicit Goldilocks principle, where all the conditions are ‘just right’ for reproduction, a range of reproductive technologies and interventions that work so as not to reproduce ‘too young’ or ‘too old’, are developed and consumed. Yet the application of assisted reproductive technologies creates new hybrid entities that bring underlying gender-age norms into question (van de Wiel 2014; Bulher 2015). These emerging hybrids are products of several RTs over time—like the ‘older mother’ who uses contraception to postpone pregnancy, ART to address age-related infertility and advanced age obstetric care for ‘late’ childbirth.

### Empty spaces

As reproductive justice reminds us, the fight for reproductive autonomy is not only about barriers to access to certain technologies, but also about who these technologies are serving and why. In this way, it also draws attention to whose needs are routinely overlooked or dismissed. A third way of considering RTs spatially is in identifying empty spaces. Work in the field of reproductive technologies has tended to foreground specific physiological events (i.e. pregnancy, childbirth, breastfeeding), specific technologies (particularly those that are more obviously technological or ‘modern’) and specific people—namely cis, white, middle-class women. This has led Adele Clarke and Haraway to claim recently that reproductive studies is “tacitly pronatalist” (2018, p. 9). While we do not necessarily agree with this characterisation based on our knowledge of our colleagues’ and our own political positions, we do think it is important to consider this implication.

Analysis within the social sciences has tended to focus on the more extraordinary or spectacular technologies. Many authors have charted the trajectory of RTs from experimental techniques at the frontier of science to being part of the standard-of-care (see Wahlberg 2016). Clarke et al. (2003) have described the same trend in biomedicine more generally, in that innovations from earlier eras become the invisible infrastructure of the next. Though some technologies may have become more mundane, they still remain significant for those who use them. Perhaps especially when technologies become normalised in this way, social scientists should be scrutinising them more closely, asking questions about what forms of reproduction they are supporting and what is excluded through their normalisation. Han’s (2013) study of contemporary pregnancy practices in the US illustrates how ‘ordinary’ and mundane RTs such as pregnancy tests and foetal ultrasounds are seamlessly incorporated into everyday practices. These technologies are near invisible, “unremarked upon because they have been perceived as unremarkable” (2013, p. 14), yet these unremarkable technologies are still culturally situated and profoundly shape women’s, and men’s, experiences (Han 2013). RTs may well move from ‘novelty to norm’ (Leavitt 2006), but earlier generations of RTs are no less engineered nor less significant in people’s lived experiences (see also Sanabria 2016; Ross 2018).

There is little research on those ‘ordinary’ technologies that are self-administered such as fertility monitoring apps, ovulation tests, and home pregnancy tests. Though there is much literature on pregnancy loss, there is less on procedures and treatments associated with preventing early pregnancy loss. While reproductive ageing has been a popular topic in studies of reproductive technologies, especially in relation to ART, there has been less attention to related hormone replacement therapy, reproductive cancer screening and treatment or associations with broader sexual health. Similarly, perhaps because of an assumption that ARTs are aimed at women who have ‘delayed’ childbirth, which itself reflects a particular way of thinking about ‘planned’ reproduction, there is little attention paid to secondary infertility. There is also little work as yet on the increasing presence of digital technologies in the reproductive process, from online platforms for information and services to social media and apps (though see Hamper 2020 for emerging work in this area).

Another important negative space to which studies of RTs could pay more attention is those technologies that have never been, or never will be, invented or developed beyond theory or prototype. We are used to periodic media reports about a breakthrough in male hormonal contraception, which seem to run out of steam just as quickly as they emerge (see Oudshoorn 2003). Some RTs, like the diaphragm or ICSI, have been popular for a while only to fade into relative obscurity, while others (female condoms, IUDs) are taken up in some places and not others. Londa Schiebinger (2004) has written about the use of botanical abortifacients amongst Indigenous people and slaves in the Caribbean and how knowledge of these plants’ use was ultimately lost in the violent encounter with European colonisers. The study of RTs parallels the development of technologies under its gaze—and certain assumptions get carried through, such as who certain technologies are aimed at and suitable for, what people’s motivations might be for using them and what constitutes ‘effective’ or ‘proper’ technologies (Martin 2012). Meanwhile, it is worth considering why more resources have not been put into preventing infertility, rather than treating it through high-impact (especially for female-gendered bodies) medical technologies. This is perhaps especially acute in a time of climate crisis, when environmental conditions are increasingly thought to affect human fertility. For example, research suggesting that air pollution has harmful effects on ovarian reserve was presented at the 2019 meeting of the European Society of Human Reproduction and Embryology (New Scientist 2019), suggesting that this may become another rationale for egg freezing (or, indeed, earlier motherhood) and a ‘growth area’ for the industry, much as this would also likely exacerbate existing stratifications and reproductive injustices. Thinking about why certain technologies do, and do not, get developed is instructive (see Hartmann 2016). It can tell us much about what kinds of knowledge and expertise are prioritised, whose needs are normalised and naturalised, and why it is that reproduction, despite coming under increasing scrutiny and surveillance, remains marginalised and feminised.

## Conclusion

To paraphrase Audre Lorde (1982), there is no such thing as single-issue reproduction, because people do not lead single-issue lives. In this article, we have argued that, while it is imperative to start from the data and to provide rich pictures of the experiences of those using reproductive technologies, we must not lose sight of the contexts in which those experiences take place, as well as how they might change over time. This means not only accounting for how reproduction happens within existing relationships, kinship models and community mores, but also the provision of services, the development of technologies themselves, the links between the fertility industry and other industries (and indeed capitalism in general), government policies, cultural, ethical and religious assumptions about how to conceptualise and treat medical problems and so much more.

The study of RTs has its own life course, context and events that have accrued and so we have argued this is an appropriate point in time to examine the lenses we use to reflect on what precepts have built up around the field and to encourage an integrated approach. More recently, renewed and increasing attention has been paid to stratified reproduction and reproductive injustice, with the voices of scholars of colour, LGBTQ+ and Indigenous scholars finally starting to receive more attention. This is vital to having a fuller understanding of reproduction and RTs. This reflects a shift, also, in who is able to enter academia and get published—the academic centres of power are, still, in the Global North and white, undoubtedly benefiting from often unrecognised privilege—but, however overdue, the lessons of intersectional theory, black feminist thought, decolonial scholarship, queer studies and reproductive justice are starting to filter through. The question of who is able to have a seat at the table in studies of RTs remains an important part of the story—as it is in any academic discipline. Is it also worth noting that the focus in academia on individual researchers militates against a sense of building a collective field for which we all bear some responsibility? Initiatives like the special issue edited by Valdez and Deomampo (2019) cited earlier, which helpfully includes a description of how the project came into being and how the core team navigated the power dynamics of their different identities, are an exciting example of what the future holds for studies of RTs. As such examples show, studies of RTs must continue to be driven by a combination of compelling data, feminist methodologies and thoughtful analysis which is relentlessly focussed on addressing the intersecting inequalities and stratifications that structure lived experiences.

## Footnotes

1 There are several topics that we recognise we could say more about—for example, the anthropological literature that urges a critical approach to demographic studies of fertility; technologies of infant feeding; work on disability and reproduction and emerging scholarship around the role of digital platforms and big data in reproduction, to name but a few.
